# Hospitalized Patients with Pandemic (H1N1) 2009, Kenya

**DOI:** 10.3201/eid1709.100992

**Published:** 2011-09

**Authors:** Eric Mogaka Osoro, Penina Munyua, Philip Muthoka, Solomon Gikundi, M. Kariuki Njenga, Samwel Lifumo, Racheal Achilla, Lilian Waiboci, Charles Nzioka, Jared Omolo, Daniel R. Feikin, Robert F. Breiman, Mark A. Katz

**Affiliations:** Author affiliations: Ministry of Public Health and Sanitation, Nairobi, Kenya (E.M. Osoro, P. Muthoka, C. Nzioka, J. Omolo);; Kenya Medical Research Institute, Nairobi (P. Munyua, S. Gikundi, M.K. Njenga, S. Lifumo, R. Achilla, L. Waiboci, D.R. Feikin, R.F. Breiman, M.A. Katz);; US Centers for Disease Control and Prevention–Kenya, Nairobi (P. Munyua, S. Gikundi, M.K. Njenga, L. Waiboci, D.R. Feikin, R.F. Breiman, M.A. Katz);; US Army Medical Research Unit–Kenya, Nairobi (R. Achilla)

**Keywords:** influenza, pandemic (H1N1) 2009, hospitalized patients, viruses, Kenya

## Abstract

To describe the epidemiology and clinical course of patients hospitalized with pandemic (H1N1) 2009 in Kenya, we reviewed medical records of 49 such patients hospitalized during July–November 2009. The median age (7 years) was lower than that in industrialized countries. More patients had HIV than the general Kenyan population.

Since pandemic (H1N1) 2009 influenza virus emerged in April 2009, virus-associated hospitalizations and deaths have been reported in many countries (*1*). However, little is known about severe cases of pandemic (H1N1) 2009 in sub-Saharan Africa. We describe the epidemiology, clinical characteristics, and clinical course of the disease in patients hospitalized with laboratory-confirmed pandemic (H1N1) 2009 infection in Kenya during July–November 2009.

## The Study

Following detection of pandemic (H1N1) 2009 virus in 2 patients in California, USA, in April 2009, the Kenyan Ministry of Health intensified surveillance at 26 existing influenza sentinel surveillance sites where surveillance was being conducted for influenza-like illness and severe acute respiratory illness. An additional 29 hospitals and clinics in the country were trained to conduct surveillance for these conditions. A hospitalized case-patient with pandemic (H1N1) 2009 was defined as a patient hospitalized for >24 hours with acute respiratory illness who had an oropharyngeal or nasopharyngeal swab specimen positive for pandemic (H1N1) 2009 virus by real-time reverse transcription PCR at either the Kenya Medical Research Institute (KEMRI)/National Influenza Center laboratory, or the KEMRI/Centers for Disease Control and Prevention, Kenya laboratory. Testing was performed according to World Health Organization methods (*2*).

We reviewed all available medical records for hospitalized patients with laboratory-confirmed pandemic (H1N1) 2009 using a standardized case report form that included demographic, clinical, laboratory, and epidemiologic information. For hospitalization duration calculation, the day of admission was hospital day 0. For patients for whom height and weight measurements were available, the body-mass index (BMI, weight in kilograms divided by the square of height in meters) was calculated. Obesity was defined as a BMI >30 in adults >18 years old (*3*).

From June 29, 2009, when pandemic (H1N1) 2009 infection was confirmed in Kenya, through November 29, 2009, 690 patients with laboratory-confirmed pandemic (H1N1) 2009 were identified. Of these patients, 88 (13%) were hospitalized in 12 surveillance hospitals. Most hospitalizations (61 [69%]) occurred during October and November (Figure). The median patient age was 5.1 years (range 1 month–61 years). Thirty-four (39%) patients were <2 years, and 19 (22%) were 18–49 years (Table).

**Figure:**
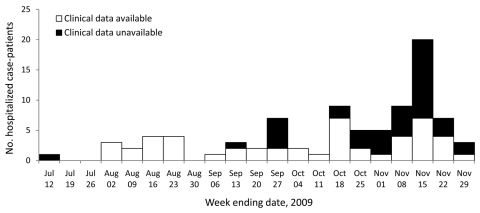
Pandemic (H1N1) 2009 case-patients hospitalized by week, Kenya, July–November 2009 (n = 88).

**Table:** Characteristics of hospitalized patients with pandemic (H1N1) 2009, Kenya, July–November 2009*

Characteristic	No. patients with characteristic/ no. with data available (%)
Female sex	46/88 (52)
Age group, y	
0–<2	34/88 (39)
2–4	9/88 (10)
5–9	10/88 (11)
10–18	13/88 (15)
19–49	19/88 (22)
>49	3/88 (3)
Underlying medical condition†	
Asthma	5/49 (10)
HIV	4/20 (20)
Obesity	4/20 (20)
Tuberculosis‡	1/25 (4)
Valvular heart disease	1/49 (2)
Oseltamivir treatment	10/42 (24)
ICU care	1/49 (2)

*ICU, intensive care unit. †Two patients had asthma and were obese. ‡In continuation phase of treatment.

We could not obtain clinical records for 39 (44%) case-patients. We describe clinical data for 49 (66%) case-patients. No significant differences were found in median age (7 years vs. 3 years; p = 0.39) or sex (51% females vs. 54% females; p = 0.40) between patients with clinical data and patients without clinical data.

The 49 case-patients were from 4 public and 3 private hospitals in 4 of Kenya’s 8 provinces. Half (51%) of case-patients were admitted to Siaya District Hospital in Nyanza Province. Thirteen (26%) patients had an underlying medical condition (Table). Of 20 patients with known HIV infection status, 4 (20%) were HIV positive. Of 20 patients with BMI information, 4 (20%) were obese. None of the adult female patients was pregnant.

The median duration between onset of symptoms and hospitalization was 3 days (range: 0–14 days). The most common symptoms at admission were self-reported fever (43 patients [88%]), cough (45 patients [92%]), and vomiting (20 patients [41%]). Eighteen (37%) patients reported diarrhea. Of 22 patients with malaria blood smear results, 5 (23%) had positive results (median patient age 5 months). Of 42 patients with data on antiviral treatment, 10 (24%) had received oseltamivir either before or during hospitalization.

The median duration of hospitalization was 4 days (range 1–51 days). All 4 HIV-infected patients survived without known complications. Two (4%) patients died. The first was a 55-year-old HIV-negative man without underlying illness who had been admitted with acute respiratory illness. He was transferred to the intensive care unit after secondary bacterial infection (*Pseudomonas aeruginosa*) developed. The patient died after 45 days while receiving mechanical ventilation in the intensive care unit. The next person who died was a 4-year-old boy without underlying illness (HIV status unknown) who had been admitted with a cough, vomiting, and fever. He had malaria confirmed by blood smear and died 4 days after admission. Neither patient received oseltamivir.

## Conclusions

This study describes the epidemiology, clinical features, and clinical course of pandemic (H1N1) 2009 infections in hospitalized patients in a sub-Saharan African country outside South Africa. The large proportion of pandemic (H1N1) 2009 hospitalizations among young adults in Kenya is consistent with findings from New Zealand, the United States, and Australia (*4**–**6*). However, the median age of 5.1 years is lower than median ages found in Australia (15 years) and the United States (20 years) (*4**,**5*). The lower median age in Kenya could be explained by varying patterns of health care use by age in Kenya. Studies in western Kenya and Nairobi have shown that adults are less likely to seek medical care at hospitals for acute respiratory illness than are young children (*7**,**8*).

One fourth of those hospitalized with pandemic (H1N1) 2009 influenza in Kenya had an underlying medical condition, a lower proportion than in the United States (73%), Ireland (50%), and Chile (37%) (*5**,**9**,**10*). This difference could be due to undetected chronic illnesses; in many health care facilities in Kenya, patients are not routinely screened for chronic diseases. Additionally, data about chronic illness may not have been recorded on hospital charts.

Twenty percent of hospitalized pandemic (H1N1) 2009 patients with available HIV data were positive for the virus. Although this percentage is higher than the national HIV prevalence (7%) (*11*), the HIV-positive patients were all from Nyanza Province, which has an HIV prevalence of 15% (*11*). Nevertheless, our findings suggest that HIV patients could be at risk for severe pandemic (H1N1) 2009 influenza, supporting results of a study in South Africa which reported that 53% of pandemic (H1N1) 2009 patients who died were HIV positive (*12*). However, our report included a relatively small number of patients who had been tested for HIV, and CD4 counts and information on use of antiretroviral drugs were unavailable.

The proportion of hospitalized pandemic (H1N1) 2009 patients who were obese (20%) is higher than the estimates of the prevalence (5%–10%) of obesity in adults in Kenya (*13*). These findings are consistent with recent studies in the United States that have shown obesity to be a risk factor for severe outcomes from pandemic (H1N1) 2009 (*14*).

Although 50,000 treatment courses of oseltamivir were available in Kenya in May 2009, oseltamivir was underused during the study period, likely because laboratory confirmation of pandemic (H1N1) 2009 was not available quickly enough to inform patient management. In addition, clinicians may not have been aware of the availability of and indications for oseltamivir. The Kenya Ministry of Health has recommended and made available oseltamivir for empirical treatment of hospitalized patients with suspected pandemic (H1N1) 2009 (*15*).

Nevertheless, our study has some limitations. Some medical charts had incomplete data and others could not be accessed. The reported cases therefore may not be representative of all hospitalized persons with pandemic (H1N1) 2009 in Kenya. In addition, reported cases were limited to hospitals where influenza surveillance was conducted and hospitals where clinicians were aware that pandemic (H1N1) 2009 testing was available. Most Kenyan hospitals have not been conducting systematic surveillance for pandemic (H1N1) 2009; thus, hospitalized patients with pandemic (H1N1) 2009 were likely missed.

Existing and enhanced sentinel hospital surveillance for influenza in Kenya made it possible to describe severe outcomes of pandemic (H1N1) 2009 in this country. Yet, the number of severe outcomes was likely much higher than reported here. Continued surveillance is essential in clarifying the full clinical spectrum and evolution of the pandemic in Kenya, including the role of coexisting conditions such as HIV infection and malaria. In addition, increasing clinician awareness of pandemic (H1N1) 2009 treatment guidelines could improve patient management**.**
